# CYR61 as a Potential Biomarker and Target in Cancer Prognosis and Therapies

**DOI:** 10.3390/cells14110761

**Published:** 2025-05-22

**Authors:** Andrew J. Schenker, Greisha L. Ortiz-Hernández

**Affiliations:** 1 Department of Medical Sciences, College of Health Sciences, Western University of Health Sciences, Pomona, CA 91766, USA; 2 Division of Biomarkers of Early Detection and Prevention, Department of Population Sciences, City of Hope Comprehensive Cancer Center, Duarte, CA 91010, USA

**Keywords:** CYR61, cancer, matricellular protein, CCN family

## Abstract

Cysteine-rich protein 61 (CYR61) is a matricellular protein in the CCN family that is involved in cellular adhesion, migration, proliferation, and angiogenesis. CYR61 interacts with integrins α6β1, αvβ3, αvβ5, and αIIbβ3 to modulate tumor progression and metastasis while modifying the tumor microenvironment. CYR61 exhibits context-dependent roles in cancer, acting as both a tumor promoter and suppressor. Increased CYR61 expression is linked to extracellular matrix remodeling, immune modulation, and integrin-mediated signaling, making it a potential prognostic biomarker and therapeutic target. Emerging research highlights the utility of CYR61 in liquid biopsies for cancer detection and monitoring. Integrin-targeted therapies, including CYR61-blocking antibodies and CAR-T approaches, offer novel treatment strategies. However, therapy-induced toxicity and resistance remain challenges with these strategies. The further elucidation of the molecular mechanisms of CYR61 may enhance targeted therapeutic interventions and improve patient outcomes.

## 1. Introduction

Cysteine-rich protein 61 (CYR61) is a member of the CCN family of matricellular proteins and has been shown to play a critical role in cellular communication, adhesion, and migration [1]. The acronym CCN represents the original members of this family: cysteine-rich protein 61, Connective Tissue Growth Factor (CTGF), and Nephroblastoma (NOV) [2]. Originally identified in 1990 as a growth factor-inducible immediate-early gene, CYR61 has since been recognized as a key regulator of angiogenesis, chondrogenesis, and fibrogenesis [3,4,5]. CYR61 interactions with integrins, heparan sulfate proteoglycans, and low-density lipoprotein receptor-related proteins enable it to modulate cell proliferation, differentiation, and immune responses [6].

**Figure 1:**
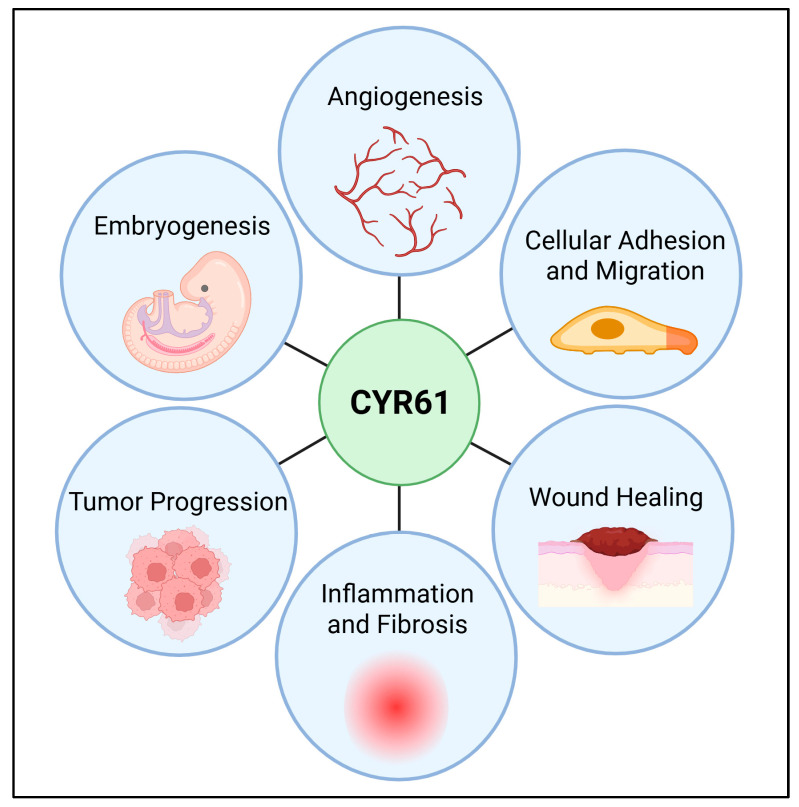
Key biological functions mediated by CYR61. The importance of CYR61 in both normal physiological processes and various pathological conditions is summarized through various biological processes. Some of the key functions of CYR61 are angiogenesis [17,25], cellular adhesion, and migration by interacting with integrins and heparan sulfate proteoglycans [19], wound healing by promoting cell proliferation and migration [26,27], inflammation and fibrosis [21,22], tumor progression implicated in tumor growth and metastasis [10,28], and embryonic development [29,30,31], particularly in the cardiovascular system. These functions highlight CYR61′s importance in both normal physiological processes and various pathological conditions. Created in BioRender. Ortiz, G. (2025) https://BioRender.com/nqb2e1j (accessed on 9 May 2025).

CYR61 consists of conserved domains that mediate its diverse biological functions, including its ability to bind integrins such as αvβ3, αvβ5 α6β1, and αIIbβ3 [2]. These interactions influence DNA synthesis, cellular adhesion, and migration, particularly in vascularized tumors and cancerous environments [7]. Describing the roles of CYR61 in cancer is crucial, as its dual functions in promoting or suppressing tumorigenesis highlight the complexity of its biological impact [8,9,10]. Indeed, CYR61 has been implicated in various cancers, including breast, prostate, pancreatic, and lung cancers, where it affects tumor progression, metastasis, and treatment resistance [11,12,13,14,15]. CYR61 has been considered relevant to cancer progression, specifically. It promotes angiogenesis by interacting with integrins and VEGFR2, facilitating the formation of new blood vessels essential for tumor growth and metastasis [16,17]. Additionally, CYR61 enhances cell migration and adhesion, contributing to the invasiveness and metastatic potential of cancer cells [18,19]. Its role in apoptosis regulation is significant, as modulating CYR61 activity can increase the sensitivity of cancer cells to apoptosis-inducing therapies [20]. Furthermore, CYR61 is involved in inflammatory and fibrotic responses, which can create a tumor-promoting microenvironment; targeting CYR61 can thus alter this microenvironment to be less supportive of cancer progression [21,22]. Lastly, CYR61 expression is linked to chemoresistance, particularly in triple-negative breast cancer, where it upregulates survivin expression and activates Wnt/β-catenin signaling, making cancer cells more resistant to chemotherapy [23] (Figure 1). These diverse functions make CYR61 a promising target for developing novel cancer therapies.

Given its involvement in multiple pathological processes, CYR61 is being explored as a potential biomarker for cancer prognosis and as a therapeutic target [24]. Understanding its molecular mechanisms could aid in the development of targeted therapies that disrupt CYR61–integrin signaling pathways. Furthermore, as research continues, CYR61′s role in immune surveillance and tissue repair further underscores its significance in both normal physiology and disease states.

## 2. Discovery

In 1990, O’Brien and colleagues in the Lau lab successfully cloned a growth factor-inducible immediate-early gene, CYR61, which encodes a 379-amino-acid polypeptide with 38 conserved cysteines, a molecular mass of 42 kilodaltons, and an N-terminal secretory signal [3,4,32]. Once associated with the extracellular matrix (ECM), CYR61′s half-life extends to greater than 24 h, and with high heparin-binding affinity, CYR61 was quickly theorized to be involved in cell-to-cell communication [6]. Bork recognized structural motifs in CYR61: CTGF and NOV. This established the designation of “CCN-family proteins”, which has expanded to encompass six members that regulate other bioactive peptides through direct binding interactions [2,32]. Kireeva and colleagues in the Lau Lab purified CYR61 and revealed its function as a chemotactic factor acting on fibroblasts, promoting cell proliferation, migration, and adhesion to endothelial cells [1,33]. As an angiogenesis-inducing ligand, CYR61 promotes cell adhesion through several binding interactions with integrins. Of the integrins with which CYR61 interacts, integrin αvβ3 augments growth factor-induced DNA synthesis and mediates the adhesion of vascular endothelial cells; integrin α6β1 binding influences fibroblast cell adhesion; and the αIIbβ3 domain can promote platelet adhesion and aggregation [7,34]. Early in vitro studies demonstrated CYR61 involvement in tissue-specific stages of chondrogenesis and, therefore, theorized that CYR61 aids in mammalian embryonic skeleton development [4,29]. By the early 2000s, CYR61 was established to be involved in fibrogenesis, angiogenesis, and chondrogenesis, as well as cell proliferation and differentiation through the direct binding of integrins, heparan sulfate proteoglycans, and low-density lipoprotein receptor-related proteins [1,30,32,35,36].

Recent findings uncovered the participation of the CCN-family proteins in regulating the production of cytokines and chemokines through autocrine and paracrine feedback and directly modifying cellular migratory processes, suggesting a pivotal role in the human immune-surveillance process [37]. Since the discovery of the roles that CYR61 plays in inflammation and tissue repair, studies have continued to explore CYR61 as a potential biomarker for therapeutic targeting and immune surveillance [24,38].

## 3. Structural Domain and Functions

As members of the CCN protein family, CYR61, CTGF, and NOV share significant sequence homology with highly conserved intron–exon regions [2,39]. The CYR61 gene has been mapped to chromosome 1p22.31 and encodes a 381-amino-acid polypeptide with 38 conserved cysteines, a molecular mass of 42 kilodaltons, and an N-terminal secretory signal [3,39,40]. The first of five exons (with four interspaced introns) encodes a secretory signal from the N-terminal, while the following four exons encode conserved mosaic CCN-family domains [19,40,41,42]. Sequence analysis of these four conserved domains reveals that they share homology with insulin-like growth factor-binding proteins (IGFBPs), the von Willebrand Factor type-C domain (vWC), the thrombospondin type-1 repeat (TSR), and the C-terminal (CT) domains of some types of collagens (e.g., collagen XIII, XXIII, and XXV) and mucins (e.g., MUC2, MUC5AC, and MUC5B) [2,42,43]. The CCN-family proteins’ conserved secretory signal, insulin-like growth factor-binding protein, vWC repeat, TSR, and CT domain regions are likely a result of exon shuffling [44]. Adhesion receptors for CYR61 include the following: αvβ3 and α6β1 with endothelial cells [45], α6β1 with fibroblasts [46], α6β1 with smooth muscle cells, αMβ2 with monocytes, and αIIbβ3 with platelets [1,5,34,45,46,47,48]. A summary of CYR61 domains, conserved sequences, binding sites, and ribbon diagrams is shown in Table 1 [40,49,50,51,52].

## 4. CYR61 Interactome

### 4.1. Integrin α6β1

**Figure 2:**
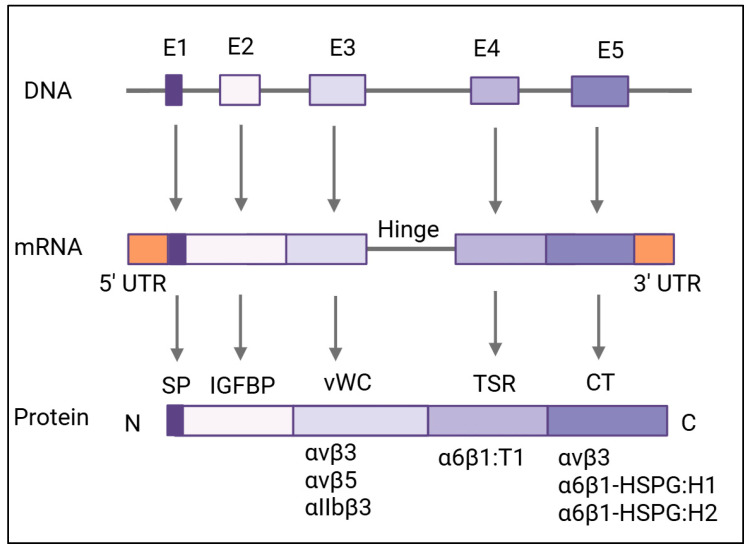
Gene and domain architecture of CYR61. The CYR61 gene undergoes transcription to produce mRNA, which is subsequently translated into the CYR61 protein. Each exon (1–5), along with its corresponding RNA transcript and protein segment, is represented as a uniquely colored rectangle. The full-length protein contains 381 amino acids with an N-terminal secretory signal peptide (SP) followed by four distinct domains. The CYR61 domains are (from N- to C-termini): the insulin-like growth factor-binding protein (IGFBP) domain, von Willebrand type-C repeat (vWC) domain, thrombospondin type-1 repeat domain (TSR), and C-terminal (CT) domain containing a cysteine-knot motif. Protein modules are labeled beneath each segment. Binding regions for integrins and heparan sulfate proteoglycans (HSPGs) on the CYR61 protein are indicated. Abbreviations: UTR—untranslated region; IGFBP—insulin-like growth factor-binding protein domain; vWC—von Willebrand factor type-C repeat; TSR—thrombospondin type-1 domain; HSPG—heparan sulfate proteoglycan; SP—signal peptide; CT—C-terminal. Created in BioRender. Ortiz, G. (2025) https://BioRender.com/g6e3mf2 (accessed on 9 May 2025).

Integrin α6β1 has binding sites in two domains of CYR61, namely, the TSR and CT domains (Figure 2 and Table 1). These sites facilitate heparin binding and integrin α6β1/heparan sulfate proteoglycan (HSPG)-mediated fibroblast cell adhesion. Specifically, within the TSR domain, the sequence GQKCIVQTTSWSQCSKS (aa 223–239) is identified as T1. In the CT domain, two sequences are identified: H1, KGKKCSKTKKSPEPVR (aa 280–295), and H2, FTYAGCSSVKKYRPKY (aa 296–314) [53]. Both the α6β1 binding domain and the cell surface HSPG binding sites work in tandem to support vascular smooth muscle cell adhesion and chemotaxis, but not chemokinesis [5]. Integrin α6β1 and HSPGs act as co-receptors in human skin fibroblasts, smooth muscle cells, and endothelial cells to mediate cell adhesion and support smooth muscle cell migration [53]. The successful binding of α6β1 and HSPGs leads to a substantial and sustained level of reactive oxygen species (ROS) and activates the cellular tumor antigen p53 and ERK/MAPK tumor suppression pathways [38,54]. Integrin α6β1 represents a promising target for antimetastatic therapies aiming to impair tumor metastasis through platelet-dependent mechanisms [55].

### 4.2. Integrin αvβ3

The binding sites for integrin αvβ3 reside within the third domain of CYR61 (Figure 2 and Table 1) and have been shown to promote pro-angiogenic activities in activated endothelial cells [44,56]. While there are several αvβ3 binding sites, Asp-125 in the 20-residue sequence of the vWC domain of V2 is particularly critical for integrin interactions [57]. Upon successful binding, downstream αvβ3-dependent pathways augment growth factor-induced DNA synthesis within the same cell type, which enables endothelial cell adhesion [7]. Binding to integrin αvβ3 allows CYR61 to promote cell proliferation, survival, and angiogenesis through the adhesion of vascular endothelial cells in a manner independent of heparin-binding activity elsewhere on the CYR61 protein [38]. The β3 class of arginylglycylaspartic acid (RGD)–integrins has α-N-(benzoxycarbonyl)-diaminopropanoic acid bundles, which contribute to the selectivity of αvβ3 over αvβ5. Although αvβ3 is typically expressed at low or undetectable levels in adults, it is involved in multiple signaling transduction pathways in cancer and tumor progression, including cell proliferation, adhesion, migration, stemness, immune escape, drug resistance, and bone metastasis. Therefore, high expression of αvβ3 in patients presents an opportunity for αvβ3-targeted therapeutics in biomarker-driven clinical trials [15]. αvβ3 expression in carcinomas such as pancreatic cancer has been shown to increase lymph node metastases in vivo and enhance anchorage-independent tumor growth in vitro [13]. Current research addressing αvβ3 antagonist toxicity reduction and limited efficacy explores a new biometric-targeted drug delivery system utilizing exosomes derived from human umbilical cord mesenchymal stromal cells (hUCMSCs) to encapsulate triptolide and generate αvβ3-specific chimeric antigen receptor T cells, both of which have been proven to induce the complete elimination of melanoma lesions [58].

### 4.3. Integrin αvβ5

CYR61 has distinct expression profiles for three non-small lung cancer (NSCLC) cell lines (H1155, H460, and H2122), five colorectal cancer cell lines (SW837, SW620, HT-29, HCA-7, and HCT116), one breast cancer cell line (MCF-7), and one esophageal squamous carcinoma cell line (TE-7) with enhanced expression of the αvβ5 integrin [11]. Integrin αvβ5 binding on CYR61 (Figure 2 and Table 1) occurs within the vWC repeat region of the second domain. The adhesion and proliferation of human breast cancer cells, astrocyte adhesion to vitronectin, and the migration of fibroblasts to CYR61 are mediated by integrin αvβ5 [44]. CYR61 tumor necrosis factor-a encounters require αvβ5, α6β1, and syndecan-4 interactions to inhibit the biphasic activation of JNK to induce apoptosis [11,59].

### 4.4. Integrin αIIbβ3

The αIIbβ3-binding site on CYR61 is within the second domain (Figure 2 and Table 1), homologous with the vWC repeat [11]. Antibodies from patients who develop thrombocytopenia post-treatment with an RGD-mimetic platelet-inhibiting drug similarly recognize ligand-inducible binding sites at αIIbβ3 [60]. The availability of pure orthosteric inhibitors of αIIbβ3 presents a tool to further research the mechanisms linking integrin conformation and deter thrombosis [61].

## 5. CYR61 Roles in Cancer

The expression of CYR61 is multifaceted and is most often associated with tumorigenesis, but can also enable tumor suppression, such as in NSCLC [14]. In certain cases, such as hepatocellular carcinogenesis, CYR61 induces pathways that generate ROS, which may both promote and inhibit tumorigenesis [62,63,64,65]. One study demonstrated that while CYR61 expression is decreased in endometrial cancer, endometrial adenocarcinoma cell lines (MDA-MB-231, AN3CA, HEC1A, HEC1B, KLE, and RL95–2) overexpressing CYR61 resulted in reduced tumor formation in nude mice [66]. This can be a result of the truncated isoform morphology of CYR61 more often having oncogenic properties, while full-length CYR61 often exhibits antiproliferative effects [41].

Somatic cells can secrete matricellular proteins into the extracellular space to join other matricellular proteins, soluble factors, and stromal cells to comprise a tumor microenvironment that is capable of the mechanical modulation of cellular activities [67]. CYR61 is highly expressed in various tumor microenvironments and can influence tumor progression by modulating the ECM to affect the adhesion, migration, and survival of cancer cells [68]. One study showed that CYR61 facilitates tumor progression in the pancreas by changing the morphology of pancreatic islets, altering the cellular microenvironment, and enabling tumor-promoting properties [69]. The expression of CYR61 in its secreted endogenous phosphorylated form is associated with aggressive metastatic phenotypes and poor prognosis in breast cancer and correlates with more advanced clinical stages, larger tumor sizes, and lymph node positivity, indicating a role in promoting tumor aggressiveness [70]. CYR61 also promotes survival in endothelial cells through integrin αvβ3 binding and induces p53-dependent apoptosis in fibroblasts through the engagement of α6β1-HSPG binding domains [53,71,72]. The increased expression of CYR61 is associated with more frequent binding of integrin avβ3, which has been shown to play a major role in breast cancer progression through the pro-angiogenic activity of tumor vascularization. Therefore, the overexpression of αvβ3 can be a biomarker for poor prognosis and a therapeutic target in breast cancer [70,73,74]. While CYR61 levels are low in healthy prostate tissue and increase during prostate cancer development within the epithelium, decreased serum CYR61 expression in patients after surgical treatment of prostate cancer is associated with a greater risk of relapse [75]. This increased expression has been shown to promote prostatic cell proliferation and, conversely, enhance the cytotoxicity of tumor necrosis factor-related induced apoptosis that selectively kills cancer cells [76,77,78]. The ambiguity of boundaries between tumors and surrounding tissue has resulted in mixed findings regarding the participation of CYR61 in different stages of various cancers [79]. Patients with ovarian epithelial carcinoma, however, had significantly higher CYR61 expression compared to patients with benign ovarian tumors, indicating a role in regional lymph node metastases and the progression of clinical disease stage [80].

CYR61 also plays a multifaceted role in hematological cancers, significantly impacting drug resistance, cell survival, and progression. Elevated levels of CYR61 in the bone marrow microenvironment of patients with acute lymphoblastic leukemia (ALL) have been shown to enhance the survival of leukemic cells [41]. Specifically, CYR61 is implicated in enhancing the survival of leukemic cells within the bone marrow microenvironment, contributing to drug resistance and poor treatment outcomes [41]. The CYR61 protein promotes cell survival and proliferation through key signaling pathways, including integrin-linked kinase (ILK) and Akt signaling, which are essential for leukemic cell growth and resistance to apoptosis [81]. Notably, CYR61′s interaction with integrins and its involvement in the modulation of the tumor microenvironment further highlight its importance in cancer biology [82]. In B-cell acute lymphoblastic leukemia (B-ALL), CYR61 has been shown to modulate chemosensitivity, with increased levels of CYR61 in the bone marrow leading to reduced sensitivity to chemotherapeutic agents such as daunorubicin (DNR) [82]. This mechanism involves the CYR61-mediated upregulation of anti-apoptotic proteins like B-cell lymphoma-2 (Bcl-2), which helps leukemic cells evade drug-induced apoptosis [82]. Additionally, CYR61 production in B-ALL cells is induced by DNA damage responses through the ataxia–telangiectasia mutated-dependent nuclear factor kappa B (NF-κB) pathway, further contributing to chemoresistance [82]. These findings underscore the potential of targeting CYR61 and its associated signaling pathways as therapeutic strategies to overcome drug resistance and improve treatment efficacy in ALL and B-ALL.

A summary of cancers associated with CYR61 domains and their respective ligands is shown in Table 2.

## 6. CYR61 in Liquid Biopsies

Liquid biopsies can facilitate the monitoring of treatment responses over time. Therefore, changes in CYR61 levels in serum may reflect the effectiveness of therapeutic interventions, allowing for the real-time assessment of patient status and adjustment of treatment plans accordingly. Liquid biopsy of serum CYR61 has potential as a diagnostic and prognostic biomarker, aiding in the detection, monitoring, and management of cancer through non-invasive means. Measuring CYR61 levels in serum presents a potentially minimally invasive and inexpensive clinical biomarker that is independent of the prostate-specific antigen and correlates with worse prognosis for colorectal cancer, breast cancer, and prostate cancer [12,54,70,83,84,85,86]. Enzyme-linked immunosorbent assays have revealed an increase in serum CYR61 levels in patients with colorectal cancer compared to patients with colorectal adenomas and healthy controls [85]. Detecting elevated serum CYR61 can improve diagnosis and decipher the clinicopathological status of patients with breast cancer [87]. In prostate cancer, higher serum CYR61 levels have been observed in patients with non-organ-confined disease compared to those with organ-confined disease, suggesting its utility in differentiating between disease stages [12]. In a study, the breast cancer mesenchymal disseminated tumor cell (mDTC) line, BC-M1, had high CYR61 levels associated with a change in microenvironmental conditions caused by viable circulating tumor cells [88].

Recently, CYR61 has emerged as a promising soluble biomarker for NSCLC, as demonstrated in a pilot study by Ackar et al. [89]. The study revealed that plasma concentrations of CYR61 were significantly elevated in patients with NSCLC compared to healthy controls, with mean levels of 13.7 ng/mL and 0.29 ng/mL, respectively. This marked difference underscores the potential of CYR61 as a diagnostic tool for NSCLC. The study further highlighted that CYR61 exhibited a sensitivity of 84% and a specificity of 100% in male patients with lung cancer, suggesting its robust performance in this subgroup. However, the sensitivity in female patients was notably lower at 27%, indicating a need for further research to optimize its diagnostic utility across different demographics. These findings support the potential of CYR61 as a circulating biomarker for the early detection of NSCLC, particularly in male patients, and warrant further investigation to validate and refine its clinical application [89].

## 7. CYR61 as a Potential Target in Cancer

**Figure 3:**
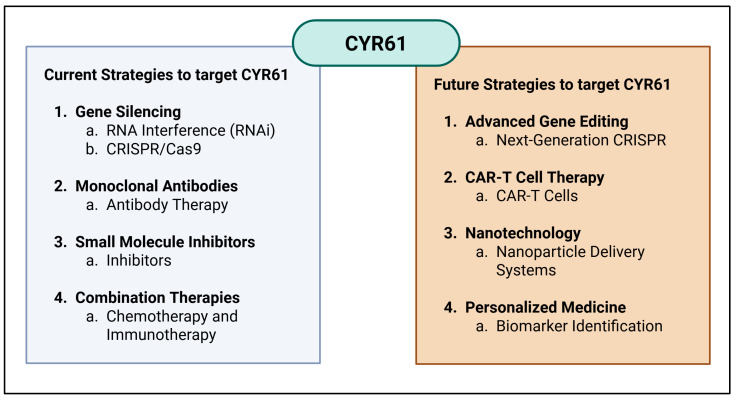
Diverse and innovative strategies developed to target CYR61 in cancer therapy and future strategies. Current strategies to target CYR61 involve gene silencing, the use of monoclonal antibodies, small-molecule inhibitors (SMIs), and a combination of therapies. For instance, gene silencing using RNAi targeting CYR61 in tamoxifen-resistant breast cancer cells decreases invasion and increases tamoxifen sensitivity. Monoclonal antibodies that block CYR61′s interaction with integrins and VEGFR2 significantly reduce tumor growth and metastasis. SMIs have shown a disruption of CYR61 receptor binding, effectively blocking cancer cell proliferation and migration. As future strategies to target CYR61, CAR-T cells engineered to target CYR61 offer a personalized treatment option for cancers with high CYR61 expression, such as triple-negative breast cancer. In addition, nanoparticles designed to deliver CYR61-targeting agents directly to tumors are a great avenue to improve drug delivery and reduce systemic toxicity. Personalized medicine approaches, including biomarker identification, enable tailored treatment plans that predict response to CYR61-targeted therapies, improving clinical outcomes. Created in BioRender. Ortiz, G. (2025) https://BioRender.com/e61pin6 (accessed on 9 May 2025).

Due to its dual role in promoting apoptosis and influencing tumor cell behavior, CYR61 may serve as a potential biomarker and therapeutic target in cancer prognosis and treatment. Modulating its activity could aid in developing strategies to enhance the efficacy of cancer therapies that rely on inducing apoptosis in tumor cells [68]. Current strategies to target CYR61 include gene silencing techniques such as RNA interference (RNAi) and CRISPR/Cas9, which effectively downregulate CYR61 expression, thereby inhibiting its pro-tumorigenic functions, like angiogenesis and cell migration [90,91]. In addition, small-molecule inhibitors that prevent CYR61 from binding to its receptors and disrupt its signaling pathways involved in cancer progression have been studied [92]. Another approach combining CYR61-targeted therapies with conventional treatments like chemotherapy or immunotherapy can enhance overall therapeutic efficacy and overcome resistance mechanisms [23] (Figure 3).

Specifically, CYR61 has been established as a critical factor in breast cancer progression, influencing tumor growth, invasiveness, and therapy resistance. CYR61 is also implicated in promoting neovascularization, as it enhances the expression of vascular endothelial growth factor (VEGF), which is crucial for tumor blood supply and growth. Cells expressing CYR61 acquire an antiestrogen-resistant phenotype, presenting a clinical challenge in breast cancer treatment [8]. This study also found that the pro-angiogenic effects of CYR61 are dependent on the VEGF/VEGF-receptor 2 (VEGF-R2) signaling pathway, and blocking this pathway with an anti-VEGF-R2 antibody abolishes the angiogenic effects of CYR61, decreasing the invasiveness of β tumors through enhanced integrin function [69]. Huang et al. identified CYR61-β1 integrin–AMPKα as a potential therapeutic target to mitigate participation in facilitating tumor cell extravasation and regulating anoikis migration of breast cancer metastasis to the lung [10]. Utilizing a blocking antibody against integrin αvβ3 is capable of inhibiting heregulin (HRG) induction of the aggressive phenotypes of breast cancer cells in vivo [8]. Because heparin is often targeted in malignant diseases for antithrombotic prophylaxis, CYR61 is a potential target to interfere with the migration of PC-3 cells [78,93]. Even though integrins can be important therapeutic targets, current RGD-based anti-integrin drugs induce conformational changes that trigger incongruous cell adhesion and potentially fatal immune reactions [94].

Future strategies to target CYR61 in cancer therapy are promising and diverse. Advanced gene editing tools, such as next-generation CRISPR systems, aim to improve the specificity and safety of gene editing for cancer therapy [95,96]. CAR-T cell therapy represents a groundbreaking approach, where T cells are genetically engineered to express chimeric antigen receptors (CARs) that specifically recognize CYR61 on cancer cells, enabling them to attack and destroy CYR61-positive tumor cells [97]. Nanotechnology offers innovative solutions, using nanoparticles to deliver CYR61-targeting agents directly to tumor sites, improving specificity and reducing systemic side effects [98]. Personalized medicine approaches, including biomarker identification, allow for more tailored and effective treatment plans by analyzing a patient’s tumor to identify unique molecular targets [99]. These strategies highlight the potential of targeting CYR61 to disrupt cancer progression and improve therapeutic outcomes (Figure 3).

## 8. Challenges

Current gaps in knowledge of the role of CYR61 in cancer include addressing integrin antagonist toxicity reduction and precisely understanding the mechanisms by which CYR61 promotes aggressive cancer phenotypes [58,100]. The complexity of integrin functions and their sometimes-opposing characteristics pose challenges in developing effective integrin-targeting therapies [101]. Cancer cells have the ability to change their integrin repertoire and become resistant to drug treatments, which may be overcome through antagonist targeting of multiple binding integrins [102].

Recently, to overcome these challenges, integrin αvβ3 CAR-T cells have emerged as a therapeutic target to halt the survival and metastasis of solid tumors such as melanoma, glioblastoma, breast, pancreatic, and prostate cancer [103]. The αvβ3 integrin is highly expressed on various tumor cells and tumor vasculature, making it an attractive target for CAR-T cell-mediated immunotherapy [104]. CYR61 plays a pivotal role in enhancing the efficacy of CAR-T cell approaches targeting the αvβ3 integrin in cancer therapy. For instance, in glioblastoma models, CAR-T cells targeting αvβ3 integrin demonstrated rapid tumor regression and prolonged survival in preclinical studies [104]. CYR61 is known to interact with αvβ3 integrin, promoting cell adhesion, migration, and survival [105]. In Schwann cells, CYR61 has been shown to regulate c-Jun expression, which is crucial for cell proliferation and migration, further highlighting its role in enhancing the therapeutic potential of CAR-T cells [105]. This interaction enhances the binding and persistence of CAR-T cells targeting αvβ3, thereby improving their cytotoxic efficacy against tumor cells [104]. Additionally, CYR61 modulates the tumor microenvironment by influencing the expression of cytokines and growth factors that support CAR-T cell function and proliferation [105]. These combined effects underscore the potential of CYR61 to augment the therapeutic outcomes of CAR-T cell therapies targeting αvβ3 integrin, offering a promising strategy for improving the treatment of solid tumors.

## 9. Conclusions

As a key member of the CCN protein family, CYR61 plays a vital role in regulating cell adhesion, migration, proliferation, and angiogenesis through interactions with integrins and heparan sulfate proteoglycans [5]. Its ability to modulate the ECM and influence tumor microenvironments has positioned CYR61 as a critical factor in both normal cellular function and pathological conditions [67]. While its role in tissue repair and immune surveillance highlights its physiological importance, CYR61′s involvement in tumor progression and metastasis underscores its dual nature in cancer biology [24,37,68]. The expression of CYR61 has been linked to both tumor-promoting and tumor-suppressive effects, depending on the cancer type and cellular context [62,63,64,65]. In cancers such as breast, prostate, and pancreatic cancer, CYR61 enhances tumor growth, invasion, and resistance to therapy, making it a promising biomarker for disease progression [8,12,13,15,102]. However, its apoptotic effects in fibroblasts and its association with tumor suppression in NSCLC indicate a more complex regulatory function [14]. Targeting CYR61–integrin interactions presents an opportunity for novel therapeutic strategies, particularly in integrin-mediated tumor progression [56,59,93,106]. Current challenges in CYR61 research include mitigating integrin antagonist toxicity and understanding the molecular mechanisms driving its pro-tumorigenic versus tumor-suppressive effects [107]. Advancements in targeted therapies, including integrin αvβ3–CAR T cells and CYR61-blocking antibodies, offer new possibilities for cancer treatment [103,105,108]. As research continues, further exploration of CYR61′s role in cancer biology and immune modulation may lead to breakthroughs in precision medicine and targeted therapy development.

## Figures and Tables

**Table 1:** CYR61 domains and binding sites.

Domain	Conserved Sequence	Binding Sites	Ribbon Diagram
Insulin-like Growth Factor-Binding Protein (IGFBP) [40,50]	TCPAACHCPL EAPKCAPGVG LVRDGCGCCK VCAKQLNEDC SKTQPCDHTK GLECNFGASS TALKGICRAQ SEGRPCEYNS RIYQNGESFQ PNCKHQCTCI DGAVGCIPLC PQELSLPNLG CPNPRLVKVT GQCCEEWVCD EDSIKDPMED QDGLLGKELG FDASEVELTR NNELIAVGKG SSLKRLPVFG MEPRILYNPL	Insulin-like growth factor-binding proteins	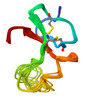
von Willebrand Factor Type-C Repeat (vWC) [51]	GQKCIVQTTSWSQCSKS	αvβ3 αvβ5 αIIbβ3	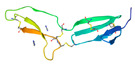
Thrombospondin Type-1 Repeat (TSR) [52]	GQKCIVQTTSWSQCSKS	α6β1	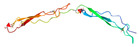
C-Terminal (CT) [2,42]	KGKKCSKTKKSPEPVRFTYAGCSSVKKYRPKY	αvβ3, α6β1-HSPG:H1, α6β1-HSPG:H2	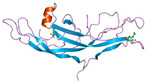

**Table 2:** Cancers associated with CYR61 binding domains and corresponding ligands.

Ligand	Binding Domain	Associated Cancers
α6β1 [38,53,54,55,71,72]	TSR, CT	Breast, ovarian, lung, lung metastasis, and prostate.
αvβ3 [13,15,38,44,56,58,70,73,74]	vWC	Bone metastasis, breast, cervical, colon, melanoma, non-small-cell lung, ovarian, glioblastoma, prostate, and pancreatic.
αvβ5 [11,44,59]	vWC	Breast, colorectal, gastric, liver metastasis, ovarian, glioblastoma, pancreatic, and prostate.
αIIbβ3 [7,34,60,61]	vWC	Breast, ovarian, and prostate.

## Data Availability

No new data were created or analyzed in this study.

## Abbreviations

The following abbreviations are used in this manuscript:

CAR-T	Chimeric Antigen Receptor T cells
CCN	Cysteine-Rich Protein, Connective Tissue Growth Factor, Nephroblastoma
CT	C-Terminal
CTGF	Connective Tissue Growth Factor
CYR61	Cysteine-Rich 61
ECM	Extracellular Matrix
HRG	Heregulin
HSPG	Heparan Sulfate Proteoglycan
hUCMSC	Human Umbilical Mesenchymal Stromal Cells
IGFBP	Insulin-like Growth Factor-Binding Protein
mDTC	Mesenchymal Disseminated Tumor Cell
NOV	Nephroblastoma
RGD	Arginine, Glycine, Aspartic Acid
ROS	Reactive Oxygen Species
TSR	Thrombospondin Type-1 Repeat
VEGF	Vascular Endothelial Growth Factor
vWC	von Willebrand Factor Type-C Domain
